# Different facets of COVID-19-related stress in relation to emotional well-being, life satisfaction, and sleep quality

**DOI:** 10.3389/fpsyg.2023.1129066

**Published:** 2023-04-14

**Authors:** Christina Saalwirth, Bernhard Leipold

**Affiliations:** Department of Psychology, Developmental and Health Psychology Unit, Universität der Bundeswehr München, Neubiberg, Germany

**Keywords:** COVID-19, financial worries, loneliness, social isolation, fear

## Abstract

**Introduction:**

As the COVID-19 pandemic has shown, it is of great importance to investigate how people can maintain their mental health during chronically stressful times. This study therefore investigated which facets of COVID-19-related stress (Fear of COVID-19, financial worries, and social isolation) impacted people the most during a third COVID-19 infection wave from March until May 2021 and how these facets relate to well-being (emotional well-being and life satisfaction) and sleep quality.

**Methods:**

A study sample of 480 German participants (*M*_age_ = 43, *SD*_age_ = 13.7, 20–69 years, 50.8% female) completed a cross-sectional online questionnaire.

**Results:**

As predicted, social isolation was reported most often, followed by fear of COVID-19 and financial worries. In accordance with our expectations more social isolation and financial worries predicted lower emotional well-being and sleep quality. In contrast to our hypothesis, fear of COVID-19 only predicted emotional well-being and not sleep quality. Life satisfaction was solely predicted by financial worries and not by social isolation and fear of COVID-19, which only partly confirmed our hypotheses. These associations remained stable after controlling for age, gender, household income, and living alone.

**Discussion:**

Financial worries, although reported the least often, were the strongest and most stable predictor for emotional well-being, sleep quality, and life satisfaction. Implications for future research and practice are discussed.

## 1. Introduction

With the beginning of the COVID-19 pandemic in 2019/2020, the lives of many people around the globe changed drastically. New challenges, fears, and restrictions arose, which constituted an ongoing source of stress. Various negative outcomes such as higher depression rates (Pieh et al., 2020), lower well-being (Zacher and Rudolph, 2021), and worse sleep quality (Mandelkorn et al., 2020), were observed in numerous countries which were driven by multiple new stressors that came along with the COVID-19 pandemic and attempts to confine infection rates to a minimum (e.g., contact bans, closing of nonessential companies and stores, and mandatory home office). Worrying about one’s financial situation (Park et al., 2020; Statista, 2020), social isolation, or uncertainty about how long social distancing requirements would be in place, were frequently reported at the beginning of the COVID-19 pandemic (Groarke et al., 2020; Park et al., 2020). However, one of the most prominent sources of stress was the fear of oneself or, even more so, loved ones contracting COVID-19 (American Psychiatric Association, 2020; Hetkamp et al., 2020; Statista, 2020; Bendau et al., 2021). Whether these facets of COVID-19-related stress are still present approximately 1 year after the SARS-CoV-2 outbreak will be investigated in this study. In addition, some studies suggested a negative relationship between COVID-19-related stress and people’s well-being and sleep quality (Cellini et al., 2020; Zacher and Rudolph, 2021), yet little is known about which aspects of COVID-19-related stress impact people the most and how they differentially relate to well-being and sleep. To close this research gap, we further examine the relationship between various measures of well-being and sleep quality with three diverse facets of COVID-19-related stress, namely fear of COVID-19, financial worries, and social isolation.

### 1.1. COVID-19-related stress

The first wave of COVID-19 infections in Germany was characterized by a great deal of uncertainty about the novel disease, strict lockdown restrictions, reduced social contacts, and frightening media content. Consequently, fear of COVID-19 was a common phenomenon. In a study from Bäuerle et al. (2020) with over 15,000 German residents, 59% of the study sample reported experiencing fear of COVID-19. Other studies have reported an increase in virus anxiety during the first wave (Jungmann and Witthöft, 2020), as well as a decrease in quality of life during the first months of the pandemic (Dale et al., 2022).

However, a rapid initial increase in fear of COVID-19 is an expected reaction. Fear is an important emotional response to new, ambiguous, and threatening stressors which often fosters adequate behavior (Lazarus, 1991). For example, increased fear of COVID-19 was associated with positive health behavior such as wearing a medical face mask, regularly washing hands, social distancing, or a positive attitude toward vaccination (Harper et al., 2020; Bendau et al., 2021; Knowles and Olatunji, 2021). Nevertheless, fear is predominantly associated with avoidance and escape tendencies and can also have negative effects on peoples’ well-being if it chronically manifests (Lazarus, 1991). Interestingly, a study by Hetkamp et al. (2020), in line with the results of the aforementioned studies, found elevated levels of fear of COVID-19 during a period of 50 days in March and April 2020, yet fear of COVID-19 decreased to initial levels before the lockdown restrictions within only 6 weeks. These results are also supported by Skoda et al. (2021), who found a strong increase in COVID-19-related fear, which rapidly decreased even below initial levels. Bendau et al. (2021) replicated these results, with fear of COVID-19 decreasing from the end of March until the middle of June in 2020. These findings demonstrate that the population displayed a habituation to the novel and threatening stressor of COVID-19 within only a few weeks, which is in line with previous research showing that people feel less anxious when they are continually exposed to a stressor (Diener and Diener, 1996; Suh et al., 1996). Even though, the opposite effect can occur as well (Ritter et al., 2016).

Whereas fear of COVID-19 was frequently reported during the COVID-19 pandemic, it is far from being a homogenous construct and can comprise many different aspects, such as a fear of getting sick, worries about insufficient food supplies, or the fear of close contact with foreigners. How multilayered fear of COVID-19 was defined by previous research can easily be seen by examining the various questionnaires that were developed to measure fear of COVID-19, such as the COVID Stress Scales (Taylor et al., 2020), the Fear of COVID-19 Scale (Ahorsu et al., 2020), or the Fear of the Coronavirus Questionnaire (Mertens et al., 2020). However, when Mertens et al. (2021) investigated several different questionnaires, a core element “fear of health” crystallized which was found in all the investigated questionnaires. For example, “fear of health” was represented by the subscales “contamination” and “danger” in the COVID Stress Scales (Taylor et al., 2020). It seems that “fear of health” embodies the underlying key component, which is why it should particularly be considered when examining fear of COVID-19.

In addition to fear of COVID-19, people also frequently reported being impacted by the restrictive policies during the pandemic regarding their social interactions. Due to the quarantine, the restriction of social contacts, and working from home everyone’s social life was impacted heavily, which can result in a feeling of loneliness. For example, in a study by Groarke et al. (2020) 27% of the British study sample reported experiencing loneliness during the first lockdown. Such changes in social routines can also lead to stress and worries when social support and sources of joy and happiness cease. In a study by Park et al. (2020), changes in social routines and uncertainty about how long social distancing requirements would last were the COVID-19 stressors that were reported most often (yet not as the most stressful). Although social isolation was apparently a very common facet of stress during the pandemic, it has gained less attention than fear of COVID-19 in previous research.

In addition to social isolation, people were also worried about the economic stability of their countries as well as their own financial situation at the beginning of the pandemic (American Psychiatric Association, 2020; Park et al., 2020; Statista, 2020). This worry was presumably driven by either the actual loss of one’s job or the fear of losing it in the near future. For example, in Germany, the number of people in short-time work increased drastically with the beginning of the pandemic (Bundesagentur für Arbeit, 2020). Overall, the financial situation of many people worsened during the course of the pandemic (Ma et al., 2022). Considering that the rate of unemployment in Germany in April 2020 increased by 18.6% compared to the previous year, this worry seems to be a comprehensible reaction (Bundesagentur für Arbeit, 2020).

With the pandemic, many novel forms of stress (e.g., the fear of COVID-19, social isolation, and financial worries) emerged that the population had to deal with, but not all of these aspects of stress affected people in the same way. How stressors caused by the pandemic are experienced depends partly on the characteristics of the specific stressor itself, but also on how this stressor is appraised by the individual, as described by the transactional model of stress (Lazarus and Folkman, 1984). With respect to fear of COVID-19, social isolation, and financial worries differences in the population’s stress appraisal can therefore be expected, as previous research suggests (Park et al., 2020). Social restrictions due to lockdown measures basically impact everyone’s life, in contrast to financial worries, which only affect a smaller portion of the population (Park et al., 2020) and fear of COVID-19, which is more strongly experienced in specific parts of the population (e.g., people with pre-existing illnesses, Bendau et al., 2021). We therefore expect a lower degree of financial worries and fear of COVID-19 than of social isolation.

### 1.2. Relations to well-being and sleep quality

Whether certain facets of stress are present in a population, though, does not indicate anything about how these facets of stress are related to general indicators of mental health, such as subjective well-being and sleep quality. Subjective well-being comprises, according to (Diener, 1984), an emotional and a cognitive evaluation of one’s present state, both of which will be investigated in this study. We always refer to both constructs when using the term “well-being” in the following text. As numerous research has reported, both well-being (Sønderskov et al., 2020 Zacher and Rudolph, 2021) and sleep quality (Blume et al., 2020; Hetkamp et al., 2020) declined in the population in the beginning of the pandemic. This is likely a result of the increased psychological stress caused by a multitude of novel stressors. Furthermore, several studies have confirmed a negative relationship between COVID-19-related stress and people’s well-being (Horesh et al., 2020; Umucu and Lee, 2020 Zacher and Rudolph, 2021) and sleep quality (Cellini et al., 2020). However, most of the research investigating COVID-19-related stress did not distinguish between different facets of stress and their specific associations with indicators for positive adaptation, such as well-being and sleep quality.

A few studies have provided evidence for significant relationships between the stress facet fear of COVID-19, well-being, and sleep. For example, Duong (2021) reported lower life satisfaction and increased sleep disturbances for people with higher levels of fear of COVID-19, and Taylor et al. (2020) reported fear of COVID-19 to be associated with higher depression scores, which can be seen as an indicator of lower well-being (Rapaport et al., 2005).

Social isolation, as another facet of pandemic-related stress, showed a relationship to sleep quality and well-being as well. For example, Horesh et al. (2020) found loneliness during the pandemic to be related to higher levels of psychological distress and lower levels of quality of life. Voitsidis et al. (2020) reported a relation between loneliness and sleeping problems. Furthermore, high social support was associated with better sleep quality whereas low social support was associated with anxiety and stress (Xiao et al., 2020). These results are very concerning considering that social restrictions were in force for several months in numerous countries and prevented people from getting social support, which has beneficial effects on peoples’ mental health and well-being (Taylor, 2011). The mental burden caused by a lack of social contact may even have increased over the course of the pandemic, unlike fear of COVID-19. The uncertainty about the pandemic and its threat potential presumably decreased the more people knew about the novel virus, whereas the loss of social support may have even become more stressful over time.

Furthermore, Pieh et al. (2020) reported a higher risk for mental health problems for people with no work or a low income, confirming a relationship between the COVID-19-related stress facet of financial worries and well-being. Besides well-being financial worries also have been associated with sleep disturbances in past research (Danielsson et al., 2016). Bearing in mind that stressors that are perceived as uncertain, ambiguous, and existentially threatening lead to fear, financial worries may have had a great impact on people’s well-being during the pandemic, especially because job insecurity was proven to be negatively associated with well-being in previous research (Witte, 1999). In addition, a study by Park et al. (2020) showed that even though financial concerns were not among the most common COVID-19 stressors (which were those related to changes in social contact), participants who experienced financial worries appraised these as extremely stressful, even more so than fear of COVID-19 and social isolation. In conclusion this means, that financial worries in the general population might not be appraised as very stressful on average, but the association with well-being and sleep might be particularly strong, because financial worries threaten people’s ability to secure a living.

Overall, even though, many studies have confirmed that different aspects of COVID-19-related stress are associated with lower well-being and sleep quality, little is known about which stressors impact people the most and how these differentially relate to their well-being and sleep quality.

### 1.3. Aims of the study

First, we aim to compare reported financial worries and social isolation to fear of COVID-19 and investigate whether these aspects of stress differ in mean values. Because COVID-19 related fear had already decreased during the first months of the pandemic (Hetkamp et al., 2020), we assume that it is appraised as less stressful than social isolation, which was among the most prominent aspects of COVID-19-related stress in previous research (Park et al., 2020). We further assume the mean value of fear of COVID-19 to be higher than the mean value of financial worries, which were only reported by a small fraction of the population in previous research (see Park et al., 2020) and should therefore not affect the general population’s stress appraisal as much as fear of COVID-19 would.

The second aim of the study is to investigate the relationships of fear of COVID-19, financial worries, and social isolation with various measures of well-being and sleep quality. Because of previous research, we assume that all investigated aspects of COVID-19-related stress correlate negatively with well-being and sleep quality. Subsequently, we also examine whether these relationships vary in strength and aim to propose a structural equation model relating all the variables to identify the most crucial facets of COVID-19-related stress in predicting people’s well-being and sleep quality. We hypothesize that social isolation and financial worries show a stronger relationship with well-being and sleep quality than fear of COVID-19, since people might have habituated to the threat of COVID-19 (Hetkamp et al., 2020) and social isolation and financial worries may have gained importance over time.

## 2. Methods

To assess current well-being, sleep quality and different aspects of COVID-19 stress participants completed a cross sectional online questionnaire that was distributed over social media. The study was approved by the appropriate ethics committee. Data collection took place in Germany from the end of March until the middle of May 2021 when COVID-19 cases and incidence rates were on the rise again (reflecting the third wave of the outbreak in Germany). The peak number of infections, with a 7-day incidence rate of 170 cases per 100,000 inhabitants, was reached at the end of April. Case numbers then slowly started to decline again (RKI, 2021). During this time, several lockdown restrictions were in place, such as a contact ban (only one contact outside of one’s own household), the closure of nonessential stores and sports facilities, mandatory home office, and a dusk-to-dawn curfew.

### 2.1. Participants

A total of 485 participants completed the online questionnaire. Participants had to be at least 18 years old; otherwise no inclusion criteria had to be met. Five participants were excluded from the analyses due to unreliable response patterns or being defined as outliers. Outliers were defined as scores above or below three standard deviations from the mean value or with a significant Mahalanobis distance (Tabachnick et al., 2007). Of the remaining 480 participants, 50.8% were female, and the mean age was 43 years (*SD* = 13.7), with a range from 20 to 69 years. Nearly one third of the study sample (30.4%) had a university degree. A total of 72.9% of the participants were currently employed, 4.8% were currently unemployed, 14% were students or in training, and for 8.3%, none of the options were representative. Regarding household income, 12.8% of the study sample earned less than 1,000€ per month (low-income earners), 58.7% earned up to 3,000€ a month, and 25.8% earned more than 3,000€ a month. The remaining 44 participants made no statement concerning their household income. Out of all the participants 24% had received at least one dose of COVID-19 vaccine, and 25.2% lived alone.

### 2.2. Measurement instruments

Apart from the descriptive statistics and control variables, we measured three different aspects of COVID-19 related stress (fear of COVID-19, financial worries, and social isolation) as well as well-being and sleep quality with the questionnaires described in further detail below.

#### 2.2.1. COVID-19-related stress

##### 2.2.1.1. Fear of COVID-19

We assessed fear of COVID-19 with the two subscales, “danger” (D) and “contamination” (C), of the established COVID stress scales (CSS) developed by Taylor et al. (2020). All items assessed the past 4 weeks. Because the two scales were highly correlated (*r* = 0.64, *p* < 0.001) and loaded on a single factor in previous research (Taylor et al., 2020) the mean score of all 12 items was calculated. The reliability of the scale was good (α = 0.92).

##### 2.2.1.2. Social isolation and financial worries

Three items assessed how strongly the COVID-19 pandemic had affected the participants’ social life during the previous 4 weeks. The items were based on the format of the CSS and can be found in Table 1. The scale ranged from 1 (*strongly disagree*) to 5 (*strongly agree*). The mean score was computed (S = Social isolation). The reliability of the scale was found to be satisfactory (α = 0.78).

**Table 1:** Items.

Scale	Item
S	I feel lonely because of the contact ban
S	Missing social interactions are stressing me
S	I spend considerably less time with my loved ones/friends
F	I am worried about my financial situation
F	My financial situation worsened due to the COVID-19 pandemic
F	I am or was dependent of financial (state) support because of COVID-19

S, social isolation; F, financial worries.

Three additional items with a 5-point Likert scale were used to evaluate financial worries (F) due to COVID-19 in the past 4 weeks. The item format again corresponded to the one of the CSS (see Table 1). The participants rated how strongly the ongoing COVID-19 pandemic had influenced their financial situation. Higher scores reflected more financial worries. The mean score was calculated. The scale showed good reliability (α = 0.85).

To investigate whether the items assessing social isolation and financial worries loaded on two separable factors a confirmatory factor analysis was conducted. The analysis confirmed a two-factor solution (*X*^2^(8) = 15.416, *p* = 0.054; CFI = 0.999, RMSEA = 0.044), which allowed a differentiation of the two facets.

#### 2.2.2. Well-being

In this study we used two measures to assess two different aspects of well-being, namely emotional well-being, which represents the emotional evaluation, and life satisfaction, which represents the cognitive evaluation of well-being.

##### 2.2.2.1. Emotional well-being

Emotional well-being was measured with the 5-item World Health Organization Well- Being Index (WHO-5). The WHO-5 is a widely used questionnaire to assess emotional well-being. Participants rated their emotional well-being for the past 4 weeks on a 6-point Likert scale with higher scores indicating higher positive affect. The mean score for all five items was calculated. The reliability of the scale was good (α = 0.89).

##### 2.2.2.2. Life satisfaction

The well-established Satisfaction with Life Scale was used to measure the participants’ overall satisfaction with life (Diener et al., 1985). The scale consists of five items, where participants rated how satisfied they are with their life on a 7-point Likert scale. A higher mean score reflects a higher satisfaction with life. Reliability of the scale was good (α = 0.89).

### 2.3. Sleep quality

To measure subjective sleep quality during the previous 4 weeks, a modified version of the subscale “subjective sleep quality” of the Pittsburgh Sleep Quality Index (PSQI; Buysse et al., 1989) was used. To increase variability the original 4-point Likert-scale was increased to an 8-point Likert-scale. Unlike in the original questionnaire higher scores indicate better sleep quality (1 = very bad, 8 = very good) to ease the interpretation of the data because higher scores also indicate better emotional well-being and life satisfaction.

### 2.4. Control variables

Age, gender, household income, living alone (yes or no), and vaccination status (vaccinated or not vaccinated) were included as control variables. Household income was measured with a Likert scale ranging from 1 to 10 (1 = a net income of less than 250€ per month, 10 = a net income of more than 4,000€ per month). Since, vaccination status did not show any significant correlations with the relevant variables (see Table 2), it was not included in the further analyses.

**Table 2:** Bivariate correlations.

	1	2	3	4	5	6	7	8	9	10	11
1. Fear of COVID-19	1										
2. Financial worries	0.19***	1									
3. Social isolation	0.19***	0.23***	1								
4. Emotional well-being	−0.20***	−0.34***	−0.32***	1							
5. Life satisfaction	−0.05	−0.39***	−0.06	0.49***	1						
6. Sleep quality	−0.13**	−0.21***	−0.18***	0.48***	0.34***	1					
7. Age	0.08	0.05	−0.14**	0.16***	−0.09	−0.06	1				
8. Gender	−0.10*	0.10*	−0.11*	0.03	−0.14	0.10*	−0.03	1			
9. Household income	−0.04	−0.29***	−0.12*	0.15**	0.23***	0.11*	0.14**	0.13**	1		
10. Living alone	0.06	−0.00	0.76	0.09	0.16**	0.6	−0.01	−0.07	0.16***	1	
11. Vaccination status	−0.07	0.03	0.04	−0.07	−0.02	−0.00	−0.18***	0.03	−0.04	−0.09*	1

*N* = 480 for all correlations except for household income (*N* = 436) due to missing data; gender: 1 = female, 2 = male; living alone 1 = yes, 2 = no; vaccination status 1 = vaccinated (partially or fully), 2 = not vaccinated; *p < 0.05, **p < 0.01, ***p < 0.001.

### 2.5. Statistical analysis

To examine the first aim of the study, we conducted a repeated-measures ANOVA with a Bonferroni-adjusted *post-hoc* analysis with a significance level of *p* < 0.05 to test whether the three aspects of COVID-19-related stress (fear of COVID-19, social isolation, and financial worries) differ in mean values. Since all three constructs were measured with the same response (5-point-Likert scale) and a similar question format, we directly compared the mean values without any transformation of the original data.

The relationships between the three facets of COVID-19-related stress (fear of COVID-19, social isolation, and financial worries), well-being, and sleep quality, representing the second aim of the study, were first investigated *via* bivariate correlations with a significance level of *p* < 0.05. Secondly, we further calculated a structural equation model (SEM) to test whether the associations between one facet of COVID-19-related stress, well-being and sleep quality remained significant after controlling for the other facets of COVID-19-related stress. The structural equation model was based on our hypotheses and the bivariate correlations. The two well-being measures (emotional well-being and life satisfaction) and sleep quality served as the criteria that were predicted by the three facets of COVID-19-related stress (fear of COVID-19, social isolation, and financial worries). The predictors and criteria were allowed to covary. Analysis was based on maximum likelihood estimates. All variables were modeled as latent variables, which were predicted by the corresponding items, except for fear of COVID-19, which was predicted by the respective means of the “danger” and “contamination” scales. To test whether the results remained stable after controlling for the control variables we included age, gender, household income, and living alone in the model. All control variables were significantly correlated with one or more well-being measures (see Table 2). The control variables were defined as additional predictors for emotional well-being, life satisfaction, and sleep quality and were allowed to covary with each other and the facets of COVID-19-related stress. The analysis was based on the full information maximum likelihood (FIML) method which allowed us to estimate parameters in the presence of missing data in the household income variable.

All calculations were conducted using JASP, version 0.14.1, and RStudio, version 1.3.1093. The descriptive statistics of all relevant variables are shown in Table 3.

**Table 3:** Descriptive statistics.

	*M*	*SD*	Min	Max	Skewness	Kurtosis
Fear of COVID-19	2.71	0.93	1.00	5.00	0.02	−0.58
Financial worries	1.88	1.11	1.00	5.00	1.24	0.51
Social isolation	3.43	1.11	1.00	5.00	−0.35	−0.74
Emotional well-being	3.64	1.08	1.40	6.00	−0.27	−0.88
Life satisfaction	4.83	1.30	1.00	7.00	−0.73	−0.15
Sleep quality	5.10	1.73	1.00	8.00	−0.44	−0.65

*N* = 480.

## 3. Results

### 3.1. Facets of COVID-19-related stress

The repeated-measures ANOVA revealed that social isolation (S) was reported as the most stressful, followed by fear of COVID-19 (G), and worrying about one’s financial situation (*F*; see Table 3). The repeated-measures ANOVA was significant (*F*(2, 958) = 329.134, *p* < 0.001, *η*^2^ = 0.41). The Bonferroni-adjusted *post-hoc* analysis revealed that all three mean differences were significant (M(S-G) = 0.719, 95%-CI [0.690, 0.981], *p* < 0.001; M(S-F) = 1.555, 95%-CI [1.409, 1.70], *p* < 0.001; M(G-F) = 0.836, 95%-CI [0.573, 0.864], *p* < 0.001). In accordance with our assumption, the feeling of social isolation was reported to be more stressful than the fear of COVID-19 and financial worries, which were reported to be least stressful.

### 3.2. COVID-19-related stress, well-being, and sleep quality

#### 3.2.1. Correlations

The correlations of all relevant variables can be found in Table 2. Emotional well-being and sleep quality were, as hypothesized, significantly negatively correlated with all three facets of COVID-19-related stress and showed the highest correlations with social isolation and financial worries. Therefore, the more COVID-19-related stress experienced, the worse the participants’ emotional well-being and sleep quality. Yet, life satisfaction was only significantly negatively correlated with financial worries and was not related to fear of COVID-19 and social isolation; therefore, our hypotheses were only partly confirmed.

#### 3.2.2. Structural equation model

**Figure 1:**
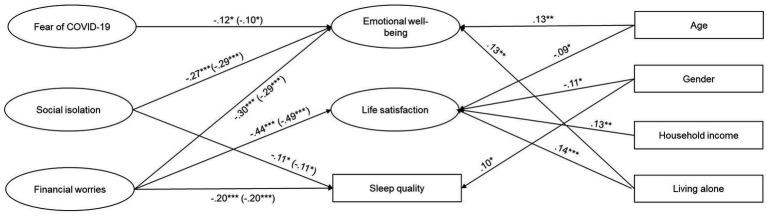
Structural equation model depicting the relationship between the three facets of COVID-19 stress, well-being, and sleep quality before and after controlling for age, gender, household income, and living alone. *N* = 480; gender: 1 = female, 2 = male; living alone: 1 = yes, 2 = no; *p < 0.05, **p < 0.01, ***p < 0.001. Only significant paths are depicted; values in brackets represent path coefficients for the model without the control variables; correlations between the predictors and control variables are not depicted for easier readability (see Table 2 for bivariate correlation coefficients).

The resulting model is depicted in Figure 1 (values in brackets on the left side of the model). A significant likelihood ratio test (*X*^2^(138) = 382.389, *p* < 0.001), a comparative fit index (CFI) of 0.951, a normed fit index (NFI) of 0.925, a standardized root mean square residual (SRMR) of 0.044, and a root mean square error of approximation (RMSEA) of 0.061 indicated an acceptable model fit. Emotional well-being was predicted by all three aspects of COVID-19-related stress, corresponding to the results of the bivariate correlations. However, the relationship with fear of COVID-19was weaker when controlling for the other two facets of COVID-19-related stress. The relationship between life satisfaction and financial worries was strengthened when controlling for fear of COVID-19 and social isolation. In addition, the associations between sleep quality and social isolation and financial worries remained significant, unlike the association between sleep quality and fear of COVID-19. Financial worries were further revealed to be a stronger predictor compared to social isolation, which showed a weakened relationship with sleep quality compared to the correlations.

The resulting model when controlling for age, gender, household income, and living alone is depicted in Figure 1. A significant likelihood ratio test (*X*^2^(190) = 514.143, *p* < 0.001), a CFI of 0.937, a NFI of 0.905, a SRMR of 0.040, and a RMSEA of 0.060 indicated an acceptable model fit. All the significant paths from the first model remained stable and significant after controlling for age, gender, household income, and living alone (see Figure 1).

## 4. Discussion

The COVID-19 pandemic and the subsequent social distancing policies led to various sources of stress during the early stages of the outbreak. Among these were fear of COVID-19, social isolation, and financial worries. This study was able to support previous research that found that social isolation and social restrictions were the most prominent aspects of COVID-19-related stress, followed by fear of COVID-19 and financial worries (Park et al., 2020; Statista, 2020), which confirmed the authors’ hypotheses. Even though people still live under an ongoing threat of getting sick, the lack of social contact appears to be far more stressful than the fear of contracting COVID-19. This might be the case because up to the date of data collection only a small proportion of the German population (approximately 4.3%, 05/15/2021) had actually suffered from COVID-19 (RKI, 2021). Therefore, the chance of becoming infected with SARS-CoV-2 was still relatively low, and people presumably had only a few or no social contacts who had already suffered from COVID-19. Hence, the restrictions of social contact reflected a more tangible negative change in people’s everyday lives than experiencing a diffuse feeling of fear of COVID-19. Furthermore, social restrictions impacted the whole population, including people who did not feel threatened by the virus, either because they did not fear getting infected or because they did not believe in its existence. This is supported by the findings of Park et al. (2020) who found that changes in social routines and uncertainty about how long social distancing requirements would last were the most prominently reported stressor in relation to the pandemic.

Nevertheless, fear of COVID-19 was still moderately present in the German population more than one and a half years after the first cases at the end of 2019, even though fear levels rapidly dropped within the first weeks of the pandemic (Hetkamp et al., 2020). This indicates that the population might have partially, but not fully habituated to the fear of COVID-19. However, since we had no data from our study sample prior to the described data collection, we can draw no conclusions about the development of fear of COVID-19 nor can we compare our statistical data to previous findings. In contrast to fear of COVID-19, financial worries were, on average, not appraised as very stressful by the general population. This may be explained by the functional German social insurance system and the several bills that were passed by the government to support people in financial need during the COVID-19 pandemic. Therefore, it is possible that in countries without comparable financial aid, this aspect of COVID-19-related stress is a more prominent issue. This should be investigated in future research to be able to compare facets of COVID-19-related stress in different countries with different social and financial structures.

The results of this study further revealed that although financial worries were not reported to be as stressful as social isolation and fear of COVID-19 in the general population, financial worries turned out to be the strongest predictor for both well-being and sleep quality, even after controlling for the other two facets of COVID-19-related stress. In fact, it was the only facet of COVID-19-related stress that was able to predict all three positive mental health indicators (emotional well-being, life satisfaction, and sleep quality). As mentioned above, only a minority of the present sample had to fear severe financial struggles, but those who did, seemed to have a greater chance of suffering from impaired well-being and sleep quality, which is in line with previous research by Park et al. (2020) and confirms our assumption that financial worries might have a great impact on people since they threaten fundamental basic needs. Furthermore, financial worries were particularly strongly associated with lower life satisfaction. Even though acute measures such as emotional well-being and sleep quality showed a negative relationship with financial worries, the global evaluation of one’s life showed an even stronger relationship. Financial worries possibly represent an existential threat that overshadows many different aspects of peoples’ lives and cause persistent worries and fears about the security of ones’ future; this may lead to an overall feeling of dissatisfaction, and can cause mental health problems such as depression or anxiety in the long run if people are overwhelmed by it.

The same goes for a prolonged reduction of social contact due to lockdown restrictions, which was also related to lower emotional well-being and worse sleep quality even after controlling for the remaining facets of COVID-19-related stress. Social contact is an important resource when struggling with problems of any kind (Taylor, 2011), and in concordance with the transactional model of stress the lack of social support can either be a stressor, but also an impairment of an important coping strategy (Lazarus and Folkman, 1984). However, when dealing with COVID-19-related stress, seeking social support was not found to be the best coping strategy compared to meaning- and problem-focused coping in previous research (Saalwirth and Leipold, 2021). Interestingly, and in contrast to the authors’ hypotheses, fear of COVID-19, although still present in the population, was only weakly correlated with emotional well-being and was not correlated with life satisfaction and sleep quality. This is an important finding considering that the population is living with the constant threat of contracting COVID-19 for quite some time. Fortunately, it seems that in general people have found a way to deal with this specific fear and remain mentally healthy under burdensome living conditions during the COVID-19 pandemic. Furthermore, this research demonstrated that these findings are independent of age, gender and household income, and therefore people in all age groups regardless of their gender or financial status are affected in the same way. Living alone also showed no substantial effect on the described relationships, not even for the COVID-19-related stress facet of social isolation. This highlights that for people living alone, the relationships between a lack of social contact and well-being and sleep quality are luckily not increased compared to those living with one or more additional household members.

### 4.1. Limitations

Although this research can extend previous findings on different facets of COVID-19-related stress and their associations with well-being and sleep quality, it is also limited in certain ways. First, the scales used to measure social isolation and financial worries were created on the basis of the COVID stress scales (CSS). They had not been used in other studies before and thus were not validated. In addition, we expanded the original time reference in the instructions of the WHO-5 and the CSS from 2 weeks and 1 week, respectively, to 4 weeks in order to align the time frame of other scales that were use. Also, the results are based solely on cross-sectional data, and therefore, no causal relationships could be tested. Future research should address this issue with longitudinal study designs with at least two, preferably more, data points. This would also allow to address the development of the relevant variables and to further investigate possible habituation processes of fear of COVID-19. In addition, further analyses regarding specific subgroups of the population, for example, students or unemployed persons who might experience financial worries differently, should be included in future research. Furthermore, the data collection took place in Germany, and thus, the transferability of the study findings for other countries should be interpreted with caution. Also, we must point out that, at the time of the data collection, not enough vaccine was available to vaccinate the entire German population; this might have biased results regarding the vaccination status of the participants. In addition, other facets of COVID-19-related stress may exist that were not investigated, but may also be relevant. For example, it is imaginable that people are also stressed by changes in work routines and child care, mistrust or discontent with governmental policies, or social discrimination, as implied by previous research (Park et al., 2020). Consequently, we do not claim completeness regarding the facets of COVID-19-related stress and future studies should extend the knowledge about different aspects of stress and their differential impacts on population health.

## 5. Conclusion

Unexpectedly, fear of COVID-19 was not or only weakly related to peoples’ emotional health and sleep quality, unlike social isolation and financial worries. Overall, financial worries turned out to be the best and most stable predictor for well-being and sleep quality. Obviously, financial worries, as an existential threat, may be a greater risk to people’s mental health than the fear of getting sick during phases of ongoing health crises and pandemics and thus should receive more attention regarding their health-related consequences by researchers and policy-makers.

## Data availability statement

The raw data supporting the conclusions of this article will be made available by the authors, without undue reservation.

## Ethics statement

The studies involving human participants were reviewed and approved by Ethics Committee of the Universität der Bundeswehr München. The patients/participants provided their written informed consent to participate in this study.

## Author contributions

CS and BL: conceptualization, methodology, and writing – review and editing. CS: statistical analysis, writing – original draft preparation, and project administration. BL: supervision. All authors contributed to the article and approved the submitted version.

## Funding

We acknowledge financial support by Universität der Bundeswehr München.

## Conflict of interest

The authors declare that the research was conducted in the absence of any commercial or financial relationships that could be construed as a potential conflict of interest.

## Publisher’s note

All claims expressed in this article are solely those of the authors and do not necessarily represent those of their affiliated organizations, or those of the publisher, the editors and the reviewers. Any product that may be evaluated in this article, or claim that may be made by its manufacturer, is not guaranteed or endorsed by the publisher.
